# Enhancement of sensory and nutritional quality of *Sel‐roti* by the incorporation of soy flour

**DOI:** 10.1002/fsn3.2550

**Published:** 2021-09-16

**Authors:** Bipana Thapa Magar, Surendra Bahadur Katawal, Anuj Niroula

**Affiliations:** ^1^ Department of Food Technology National College of Food Science and Technology Tribhuvan University Kathmandu Nepal; ^2^ Department of Food Technology Nagarik College Tribhuvan University Kathmandu Nepal

**Keywords:** nutritional value, *Sel‐roti*, sensory perceptions, soy flour

## Abstract

*Sel‐roti* is a doughnut‐like deep‐fat fried, puffed, spongy, ring‐shaped, and fermented rice‐flour confectionary indigenous to Nepal. This study was aimed to enhance the sensory and nutritional quality of *Sel‐roti* by incorporating soy flour. The best product was selected by a series of sensory evaluations, and the nutritional quality was then compared with the control. *Sel‐roti* prepared by incorporating 10% of roasted soybean flour (RSF) had significantly superior (p < .05) sensory perceptions viz. color and appearance, texture, flavor, taste, and overall acceptance among the studied samples. The moisture, crude fat, crude protein, total ash, crude fiber, iron, calcium, and energy content in the best product were significantly increased (p < .05) by the incorporation of 10% RSF. However, total carbohydrates was significantly reduced (p < .05) at 10% RSF incorporation. The results can be used by food processors to formulate a batter for the production of *Sel‐roti* with enhanced sensory and nutritional properties.

## Graphical abstract

This study was aimed to enhance the sensory and nutritional quality of Sel‐roti by incorporation of soy flour. Sel‐roti prepared by incorporating 10% roasted soybean flour (RSF) had significantly superior (p < .05) sensory perceptions among the studied samples. The moisture, crude fat, crude protein, total ash, crude fiber, iron, calcium, and energy content in the best product were significantly increased (p < .05) by the incorporation of 10% RSF.

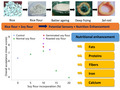

## 1 INTRODUCTION

*Sel‐roti* is a fermented rice‐flour confectionary indigenous to Nepal, which is doughnut‐like deep‐fat fried, puffed, spongy, and ring‐shaped (N. R. Dahal et al., 2005; Subba & Katawal, 2013; Yonzan & Tamang, 2009). It is prepared by deep‐frying batter made of rice flour, sugar, water, and cream/butter/ghee in ghee/oil into a ring structure (Katawal & Subba, 2008; Yonzan & Tamang, 2010). Historically, it has remained as “*Prasada*” in various Hindu rituals and festivals with specific mentions in ancient Hindu scripts like *Puran* and *Swasthani Bratakatha* (Katawal & Subba, 2008; Subba & Katawal, 2013) and is served during the marriage ceremony, various *Pooja*, and festivals like *Dasai*, *Tihar*, *Maghay Sakranti*, etc. (Yonzan & Tamang, 2010). *Sel‐roti* has remained as an ethnic fermented food of the Nepali communities in the Himalayan regions of Nepal, India, and Bhutan (Jyoti Prakash Tamang et al., 2016; Yonzan & Tamang, 2009, 2010). But, it has evolved as one of the celebrated snack foods optionally fermented and popular in all geographic regions and almost all tribes and communities of Nepal (Subba, 2012; Subba & Katawal, 2013). Snack foods consumption is on the increase due to urbanization and the food‐based industries can exploit this by developing novel snack foods with locally available flours that potentially enhance the quality of the product (Oke et al., 2018).

The information regarding the origin, ingredients and their functions, recipe, method of preparation, types of equipment, quality characteristics, and factors affecting the quality of *Sel‐roti* have been surveyed and documented previously (Katawal & Subba, 2008; Yonzan & Tamang, 2009). According to Katawal and Subba (2008), doughnut‐like ring‐shape, puffed and spongy nature, and sweet taste are indispensable characteristics of *Sel‐roti*. Other desirable attributes included golden‐brown color, grainy, glossy, and little crispy (but not hard) crust, soft and spongy (not fluffy) crumb, slightly burned/fried flavor, and oily/fatty mouthfeel (Katawal & Subba, 2008; Subba & Katawal, 2013), which is similar to other deep‐fried products and an intermediate to cake‐type (without yeast) and risen‐type (with yeast) doughnuts (Gertz, 2014; Oke et al., 2018; Vatankhah et al., 2017). *Sel‐roti* is also praised for its high storage life of about two weeks at room temperature and its nutrient density (Yonzan & Tamang, 2009, 2010). *Sel‐roti,* as a fermented product is expected to have high protein quality as compared to nonfermented rice products. Fermentation of cereals including rice results in a significant increase in water‐soluble nitrogen (Yonzan & Tamang, 2010) and specifically the limiting amino acid “lysine” (Hamad & Fields, 1979). But, considering the limiting presence of lysine in rice but richness in methionine, incorporation of soybean, which is deficient in methionine but is rich in lysine could potentially enhance the protein quality (Balasubramanian et al., 2012) of *Sel‐roti*. Roasting and germination of soybean enhance the digestibility and bioavailability of nutrients of the product as compared to normal soybean (Agume et al., 2017; Dikshit & Ghadle, 2003). It is, therefore, roasted or germinated soy flour could be a better option to incorporate in *Sel‐roti*, but further study of sensory attributes is essential.

With the increasing popularity and demand of *Sel‐roti* as a delicious energy‐dense food with intermediate shelf‐life, *Sel‐roti* is commercially available in both packed and unpacked form (S. Dahal & Katawal, 2014; Subba, 2012; Yonzan & Tamang, 2010). Products with delicious flavors, ready‐to‐eat, good nutritional quality, and availability in different varieties at an affordable cost have higher consumer preference and consumption (Subba, 2012; Vatankhah et al., 2017). However, the scientific study of *Sel‐roti* is limited to a handful of studies on microbiology, the particle size of flour, and some sensory aspects were reported (S. Dahal & Katawal, 2014; Subba & Katawal, 2013; Yonzan & Tamang, 2010). All other publications about *Sel‐roti* were limited to the introduction of the product, origin, or history (N. R. Dahal et al., 2005; Katawal & Subba, 2008; Subba, 2012; Jyoti P. Tamang et al., 1988; Yonzan & Tamang, 2009). This study is one of (if not) the first scientific reports on the enhancement of sensory and nutritional quality of *Sel‐roti*. With all the above remarks, this study was aimed to study if the sensory and nutritional quality of *Sel‐roti* could be enhanced by the incorporation of soy flour. Also, the study was carried out to evaluate if roasted soy flour (RSF) and germinated soy flour (GSF) could be used as a better alternative to normal soy flour (NSF). Lastly, the food value of the best formulation in this study was compared with control *Sel‐roti* prepared without the incorporation of soy flour.

## 2 MATERIALS AND METHODS

### 2.1 Raw materials

All raw materials were procured from Kathmandu, Nepal. *Sarana Mansuli* rice (1‐year‐old paddy: *Oryza sativa* L.), *Nepali bhatmas* (1‐year‐old, brown variety soybean: *Glycine max* L.), sugar, and refined soybean oil were purchased from the local vendor of Asan, Kathmandu. Ghee produced by Dairy Development Corporation (DDC), Kathmandu, Nepal was used.

### 2.2 Preparation of rice and soy flour and their mix

Rice was soaked in clean water for 4 hr before milling. The flour was obtained by milling in an electric grinder and sieved. The milled rice flour was separated into three parts, coarse (>890 µ), medium (225–450 µ), and fine (<120 µ). The milled soy flour of medium particle size (225–450 µ) was referred to as normal soy flour (NSF) and was primarily used for incorporation in *Sel‐roti*. As an alternative to NSF, roasted soy flour (RSF) and germinated soy flour (GSF) were also prepared. RSF was prepared by roasting soybean at 130 ± 5℃ for 120 ± 10 s and cooling to room temperature before grinding. GSF was prepared by milling germinated and dried soybeans. For germination, soybean was soaked for an hour and germinated for 3 days in the dark at room temperature with intermittent water spray 3 times a day. The germinated soybeans were dried at 40 ± 2℃ for 24 hr before milling.

Rice‐flour mix in the proportion of 30 parts coarse (>890 µ), 50 parts medium (225–450 µ), and 20 parts of fine (<120 µ) was used as control (Subba & Katawal, 2013). The incorporation of different soy flour was carried out by equivalent substitution of medium‐sized rice flour.

### 2.3 Preparation of *Sel‐roti*

**FIGURE 1:**
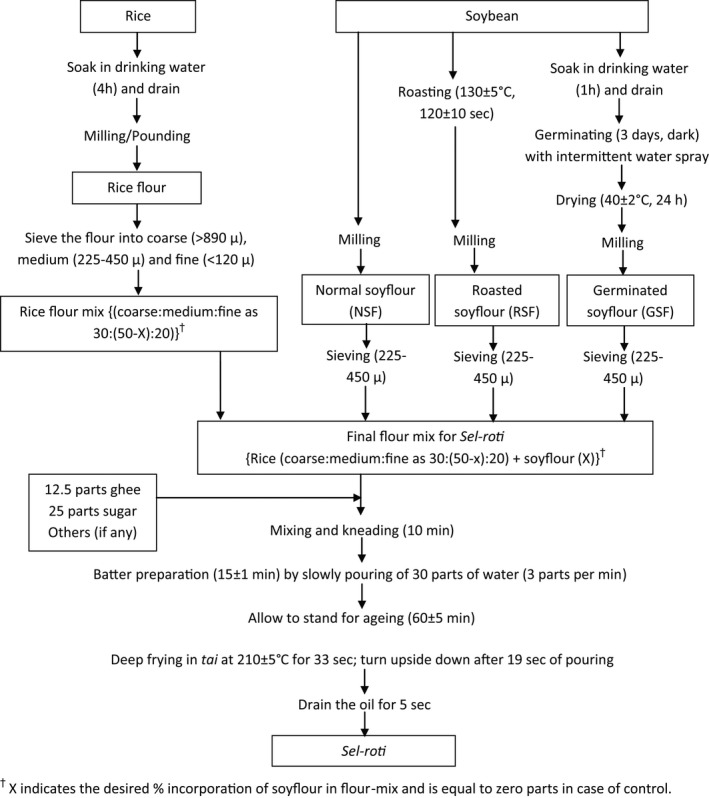
Flow‐sheet for the preparation of *Sel‐roti* preparation

*Sel‐roti* was prepared according to the procedure mentioned by Subba and Katawal (2013) with minor modifications as shown in Figure 1. Briefly, 12.5 parts of ghee and 25 parts of sugar were added to 100 parts of the flour mix and kneaded properly. 30 parts of water were poured slowly with continuous kneading and mixing to obtain a battered state. Batter preparation was completed in 15 ± 1 min. The batter was then allowed to stand for 60 ± 5 min for aging. Aged batter (37.5 ± 1g) was poured through ladle in ring shape into frying pan (locally referred to as *Tai*) with soybean oil at 210 ± 5℃. With the help of a bamboo stick (locally referred to as *Suiro*), the frying mass was turned upside down after 19 s and removed out of frying oil after a total frying time of 33 s. Any excess oil was drained by hanging in *Suiro* for 5 s. The obtained golden‐brown ring‐structured puffed product is referred to as “*Sel‐roti*.”

### 2.4 Sensory evaluation

Sensory evaluation of *Sel‐roti* was conducted through 10 semitrained panelists (in two blocks) using a 9‐point hedonic rating (9 = like extremely, 1 = dislike extremely) for color and appearance, texture, flavor, taste, and overall acceptance. Ring shape, puffed structure, and light to golden‐brown color with grainy surface were considered as the desirable appearance attributes of *Sel‐roti*. Moderate sweet taste, slightly burnt flavor, and soft but little crispness texture were considered other desirable attributes. The overall eating experience and desire for repeated consumption were considered as the parameters for the overall acceptance of *Sel‐roti*.

### 2.5 Nutritional value

Moisture, crude fat, crude protein, crude fiber, and total ash, reducing sugar (as DE), and total sugar contents were determined as described in AOAC (2005). The protein conversion factor used for rice flour and *Sel‐roti* was 5.75 and for soy flour was 6.25. Total carbohydrate was determined by the difference method. For the determination of calcium content, the sample ash was precipitated with saturated ammonium oxalate, the precipitates were then dissolved with hot dilute sulfuric acid and then titrate with Potassium permanganate. Iron content was determined as ferric iron by treating the ash with Potassium thiocyanate in an acidic environment and by plotting the absorbance at 480 nm against the calibration curve obtained for the standard iron sample. Energy value was determined by multiplying carbohydrate, protein, and fat by 4, 4, and 9 kcal per g, respectively, and summing up.

### 2.6 Statistical analysis

The data obtained were analyzed by one‐way analysis of variance (ANOVA) and sample means were compared by Tukey‐HSD test in JMP PRO 14 (SAS Inc) software. All statistical evaluations were carried out at a 5% level of significance.

## 3 RESULTS AND DISCUSSION

### 3.1 Nutritional value of rice and soybean

The proximate composition *viz*. moisture, crude protein, crude fat, crude fiber, total ash and carbohydrates, and the minerals (iron and calcium) of rice and soybean were evaluated. The results of these evaluations are presented in Table 1. Except for carbohydrates, all evaluated parameters were significantly higher (p < .05) in soybean than in rice. The values for rice were in the comparable range of crude protein (7.74%–14.76%), crude fat (0.07%–2.17%), total ash (0.39%–1.63%), crude fiber (0.23%–1.17%), carbohydrates (83%–91.8%), iron (0.27–2.65 mg/100 g DM), and calcium (7.16–33.34 mg/100 g DM) reported in previous studies (DFTQC, 2017; Subedi et al., 2016; Verma & Srivastav, 2017). The values for soybean were in the comparable range of crude protein (33.3%–47%), crude fat (14%–22%), total ash (3.8%–5.3%), crude fiber (3.7%–6.6%), and carbohydrates (20.9%–41.5%) reported in previous studies (Agume et al., 2017; DFTQC, 2017; Gebrezgi, 2019; Joshi & Rahal, 2018; Kamboj & Nanda, 2016; Michels et al., 2016; Xiao et al., 2021). Iron and calcium content in soybean were also within the range of 7.31–10.4 mg/100 g DM for iron and 192.75–240 mg/100 g DM for calcium in previous studies (DFTQC, 2017; Kamboj & Nanda, 2016; Xiao et al., 2021).

**TABLE 1:** Nutritional composition of rice and soybean

Component	Rice	Soybean
Moisture (%)	11.83 ± 0.19^b^	13.47 ± 0.17^a^
Crude protein (% DM)	8.39 ± 0.18^b^	42.23 ± 0.84^a^
Crude fat (% DM)	1.46 ± 0.09^b^	16.45 ± 0.18^a^
Crude fiber (% DM)	0.24 ± 0.03^b^	5.48 ± 0.08^a^
Total ash (% DM)	0.73 ± 0.04^b^	5.07 ± 0.06^a^
Carbohydrates (% DM)	89.39 ± 0.32^a^	30.78 ± 1.14
Iron (mg/100 g DM)	0.45 ± 0.03^b^	7.36 ± 0.32^a^
Calcium (mg/100 g DM)	11.07 ± 0.26^b^	221.78 ± 2.65^a^

Note

Values are means of three subquarters ± standard deviations. Twenty random kernels were measured for the length and breadth from each subquarter. Values with the same superscript in a column are not significantly different (p > .05).

### 3.2 Sensory evaluation of NSF incorporated *Sel‐roti*

**FIGURE 2:**
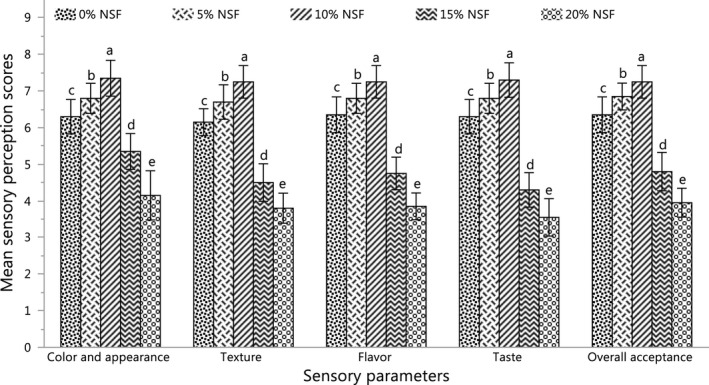
Sensory perception scores of *Sel‐roti* prepared by the incorporation of 0%–20% NSF. Each bar is the plot of the average of scores from 10 semitrained panelists in two blocks. The error bars represent the standard deviation of 20 scores. Different alphabets above the bars indicate a significant difference (p < .05) among the samples for the given parameter such that a > b>c > d>e. (NSF, normal soy flour)

Five formulations of *Sel‐roti* were prepared by incorporating (0%–20%) NSF. The results of the sensory evaluation for each evaluated parameter are presented in Figure 2.

#### 3.2.1 Color and appearance

All samples were prepared in ring shape of equal sizes. The golden‐brown crust color with a grainy surface was praised for all samples. The grainy surface was attributed to large‐sized rice‐flour particles (Subba & Katawal, 2013). The color development during frying of sugar‐rich products are primarily associated with caramelization and Maillard reactions (Bordin et al., 2013; Tamanna & Mahmood, 2015), but other factors like chemical browning, the absorption of frying oil, the density of the fried product, the temperature and frying time may also lead to color development during the frying process (Bordin et al., 2013; Loewe, 1993). The effect on the crust and color of the crumb was related to the level of soy‐flour incorporation, which is in agreement with the results reported on the effects of partial substitution of wheat flour with breadfruit flour on quality attributes of the fried doughnut (Oke et al., 2018).

The sensory score for the color and appearance significantly increased (p < .05) on increasing the level of NSF up to 10%, and thereafter significantly decreased (p < .05) at 15 and 20%. The increasing scores with the increase in NSF were possibly due to increased gloss of the crust, which could be associated with the high oil adsorption capacity of NSF as compared to rice flour (Twinomuhwezi et al., 2020). The reduced perception for *Sel‐roti* prepared by the incorporation of 15 and 20% NSF was due to their reduced puffiness and uneven crumb. When the batter is poured at extremely high heat, the entrapped moisture tries to quickly escape and create an expansion of the structure (Mcdonough et al., 2001), but when the flour with high water adsorption capacity is used, more moisture is retained in the product and thus less amount of moisture that tried to escape possibly resulted in less expansion.

#### 3.2.2 Texture

The sensory score for the texture also increased on increasing the level of NSF up to 10%, and thereafter it decreased at 15 and 20%. The increase in sensory perceptions at low levels of NSF was possibly associated with an increase in crust brittleness because soy flour is rich in both protein and fiber as compared to rice flour. The addition of protein and fiber in starch‐based fried products reduces the hardness and stiffness of the products and makes them more crispy due to the disruption of the starch matrix (Dueik et al., 2014; Surojanametakul et al., 2020).

On the other hand, *Sel‐roti* with higher levels of NSF (15 and 20%) was reported to have a less crispy crust and chewy crumb and hence received lower ratings for texture. Mcdonough et al. (2001) also reported a decrease in the crisp texture of soy flour fortified fried tortilla chips with increasing soy flour and suggested the phenomena to be associated with the increase in moisture absorption. As the level of NSF increased, the protein content of the product also increased, and hence the moisture absorption and retention capacity of a product increase with the increase in its protein content (Jideani, 2011); which possibly resulted in higher moisture retention and disrupted the balance of crisp crust and moist crumb, which probably contributed to chewy texture and reduced perception scores.

#### 3.2.3 Flavor

The perception scores for the flavor of prepared *Sel‐roti* were significantly increased (p < .05) with increasing NSF incorporation up to 10% but at a higher level of incorporation of NSF (15 and 20%), the perception scores were significantly reduced (p < .05). The flavor of wheat bread supplemented with soy flour was also reported to have similar results (Dhingra & Jood, 2002). The increase in sensorial perceptions of *Sel‐roti* at lower levels of NSF could be associated with higher content of proteins, especially Lysine; which is the first amino acid involved in Maillard reaction and thus promoting the additional formation of intermediate products of Maillard reactions called Amadori products or premelanoidins like furosine, hydroxymethyl‐furfural (HMF), and acrylamide (Bordin et al., 2013; Tamanna & Mahmood, 2015). The reduction in perception scores of *Sel‐roti* at higher levels of NSF (15 and 20%) was associated with beany notes as commented by panelists.

#### 3.2.4 Taste

All samples were moderately sweet, but the taste pleasantness was reported to be significantly different (p < .05). The sensory score for the taste significantly increased (p < .05) on increasing the level of NSF up to 10%, and thereafter it significantly decreased (p < .05) at 15 and 20%. Similar results were also reported in the taste of wheat bread prepared by soy‐flour supplementation (Dhingra & Jood, 2002). Enhancement of sensory perceptions of *Sel‐roti* prepared by incorporation of low levels of NSF (5 and 10%) was possibly due to the development of umami taste notes during the fermentation of batter. Fermentation is the economic and efficient method of producing umami taste attributes in foods like beans, grains, milk, fish, meat, and some vegetables (Zhao et al., 2019). Reduction in the perception scores of *Sel‐roti* at a higher level of NSF (15 and 20%) was associated with the introduction of bitter notes of soybean after ingestion. Soybean at higher concentrations could induce a mild bitter taste (Mohajan et al., 2018).

#### 3.2.5 Overall acceptance

The panelists were asked to provide the ratings for overall acceptance based on their overall eating experience and desire for repeated consumption. The overall acceptance scores were also significantly increased (p < .05) at low levels of NSF incorporation (5 and 10%) and significantly decreased (p < .05) at high levels of NSF incorporation (15 and 20%). The result was similar to previous attributes. Each of these attributes had a significant effect (p < .05) on the overall acceptance of *Sel‐roti*. Hence, overall acceptance of *Sel‐roti* is the combined effect of all evaluated attributes.

All evaluated parameters including overall acceptance were reported to be significantly increased (p < .05) with increasing incorporation of NSF until 10%. However, the sensory perceptions for samples decreased sharply with a further increase in NSF (15% and 20%). Therefore, the sample prepared by incorporation of 10% NSF was the best among *Sel‐roti* prepared by the incorporation of different proportions of NSF (0%–20%) based on the parameters studied.

### 3.3 Sensory evaluation of NSF, RSF, and GSF incorporated *Sel‐roti*

**FIGURE 3:**
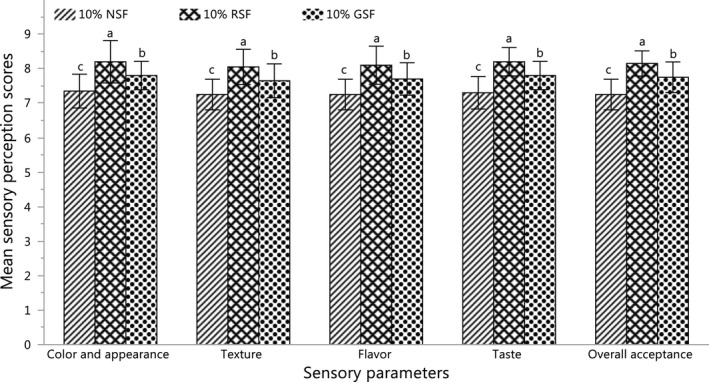
Sensory perception scores of *Sel‐roti* prepared by the incorporation of NSF, RSF, and GSF. Each bar is the plot of the average of scores from 10 semitrained panelists in two blocks. The error bars represent the standard deviation of 20 scores. Different alphabets above the bars indicate a significant difference (p < .05) among the samples for the given parameter such that a > b > c. (NSF, normal soy flour, RSF, roasted soy flour, GSF, germinated soy flour)

RSF and GSF were evaluated as potential alternatives to NSF at a 10% level of incorporation. The obtained results for each evaluated parameter are presented in Figure 3.

#### 3.3.1 Color and appearance

*Sel‐roti* prepared by the incorporation of RSF was significantly superior (p < .05) in color and appearance as compared to those prepared by the incorporation of GSF and NSF. Higher sensory scores for *Sel‐roti* prepared by incorporation of RSF and GSF were probably associated with the breakdown of protein to small moieties (Eke & Akobundu, 1993; Joshi & Rahal, 2018), which promotes Maillard browning in fried products (Bordin et al., 2013; Tamanna & Mahmood, 2015). Comparatively higher scores for *Sel‐roti* prepared by incorporation of RSF were probably due to their high oil absorption capacity and associated glossiness. Products rich in fats and oil generally have better glossiness (Gillatt, 2001). An increase in the oil absorption capacity of RSF was possibly associated with the breakdown of protein to small moieties, which unmask the nonpolar residues from the interior of native molecules (Eke & Akobundu, 1993; Joshi & Rahal, 2018; Kinsella, 1976).

#### 3.3.2 Texture

The texture of *Sel‐roti* prepared by incorporation of RSF was significantly superior (p < .05), followed by the incorporation of GSF, and the least was reported for the incorporation of NSF. This could be associated with a higher proportion of low molar mass protein in roasted and germinated flour as compared to normal flour (Joshi & Rahal, 2018; Kavitha & Parimalavalli, 2014), which reduces the hardness and stiffness of the products and makes them crispier due to the disruption of the starch matrix (Dueik et al., 2014; Surojanametakul et al., 2020). In addition, comparatively higher scores for *Sel‐roti* prepared by incorporation of RSF could also be associated with higher oil uptake property of RSF, which contributes to better mouthfeel (Eke & Akobundu, 1993; Gillatt, 2001).

#### 3.3.3 Flavor

The flavor of *Sel‐roti* prepared by incorporation of RSF was significantly superior (p < .05), followed by the incorporation of GSF, and the least was reported for the incorporation of NSF. This could be associated with the formation of different flavor components in soybean during roasting and germination followed by drying (Agume et al., 2017; Shin et al., 2013). The results could also be partly associated with enhanced contents of soluble proteins, free amino acids, and simple sugars in RSF and GSF; which promote the formation of Amadori products and premelanoidins that contribute to desirable fried flavor (Bordin et al., 2013; Tamanna & Mahmood, 2015). In addition, the higher oil absorption capacity of RSF increased the total fat content of the sample and enhanced the flavor (Eke & Akobundu, 1993; Gillatt, 2001).

#### 3.3.4 Taste

The taste of *Sel‐roti* prepared by incorporation of RSF was significantly superior (p < .05), followed by the incorporation of GSF, and the least was reported for the incorporation of NSF. This could be associated with the enhanced release of glutamic acid during roasting and germination that contributes to the umami taste. Processes like roasting, germination, and fermentation contribute to the degradation of macromolecules in foods; for example, the protein is broken into free amino acids, peptides, and nucleotides, which releases the umami component, that is, glutamic acid in them (Mouritsen, 2012; Zhao et al., 2019). In addition, the higher oil absorption capacity of RSF and GSF enhances the total fat content of the sample and enhances the taste of products (Eke & Akobundu, 1993).

#### 3.3.5 Overall acceptance

The overall acceptance of *Sel‐roti* prepared by incorporation of RSF was significantly superior (p < .05), followed by the incorporation of GSF, and the least was reported for the incorporation of NSF. Each of the above attributes had a significant effect (p < .05) on the overall acceptance of *Sel‐roti*. All evaluated parameters including overall acceptance were reported to be significantly superior (p < .05) for *Sel‐roti* prepared by the incorporation of RSF as compared to *Sel‐roti* prepared by incorporation with GSF and NSF. Therefore, the sample prepared by 10% incorporation of RSF was the best among *Sel‐roti* prepared by the incorporation of 10% of RSF, GSF, and NSF.

**FIGURE 4:**
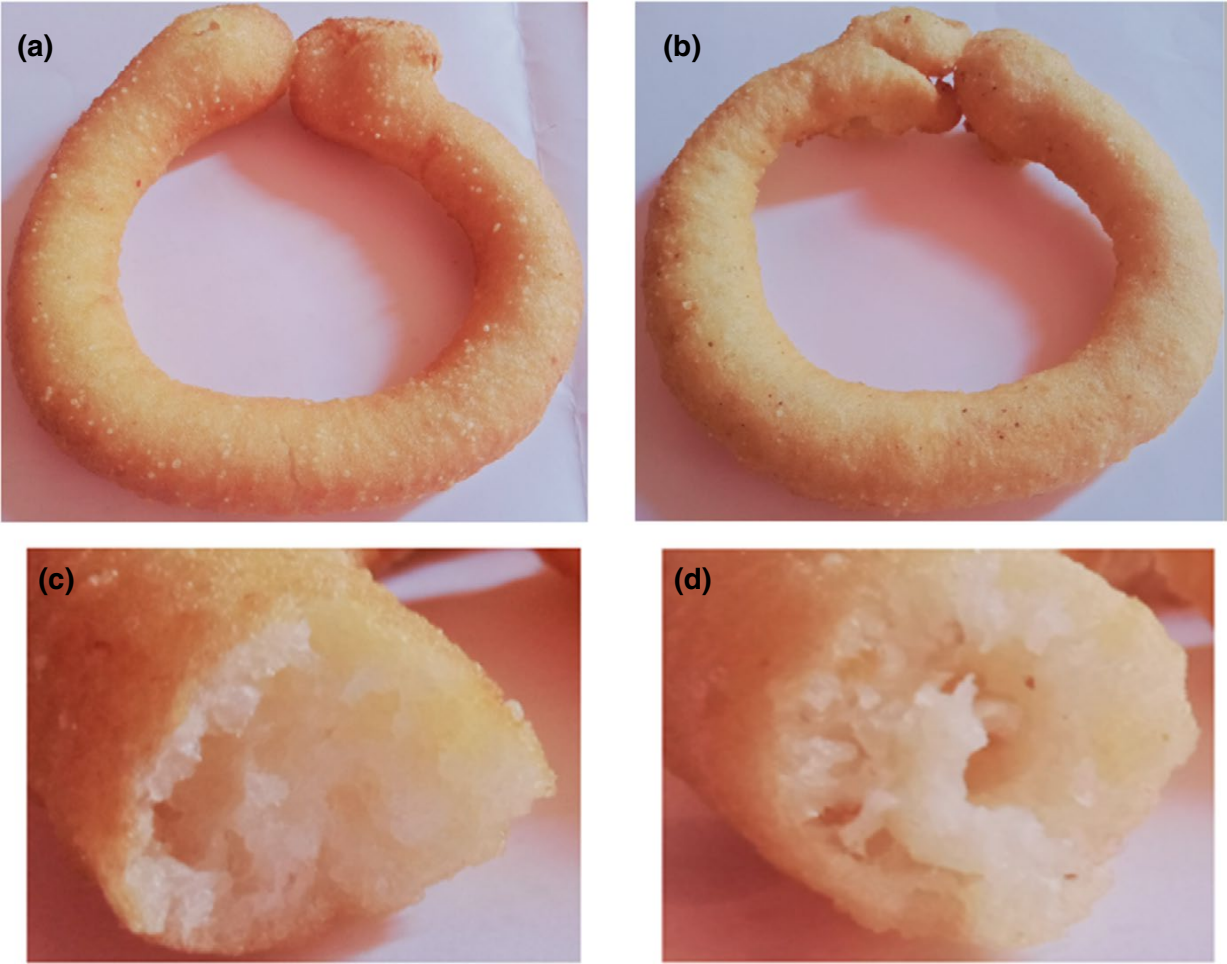
Images of control (0% RSF) (a,c) and best (10% RSF) (b,d) *Sel‐roti* samples. a, b: top view; c, d: cross‐section view

An additional sensory evaluation of *Sel‐roti* prepared by incorporation of RSF (10 and 12.5%) was carried out to determine if an additional amount of RSF could potentially increase the sensory perceptions of *Sel‐roti* and were compared with control and presented in Table S1. Sensory perceptions of *Sel‐roti* prepared by the incorporation of 12.5% RSF were significantly superior (p < .05) than control and significantly inferior (p < .05) to *Sel‐roti* prepared by incorporation of 10% RSF. Therefore, the *Sel‐roti* prepared by the incorporation of 10% RSF was best among all *Sel‐roti* samples evaluated in this study. The images of the control (0% RSF) and best (10% RSF) are presented in Figure 4. Additionally, the important physical properties of the samples viz. weight per piece, ring diameter, oil uptake, and bulk density that could significantly influence the sensory properties of *Sel‐roti* were also evaluated for the control and best product. The results for some evaluated physical properties are presented in Table S2 for better insights.

### 3.4 **Nutritional evaluation of control** and **best product**

The proximate composition, energy value, reducing sugar (as dextrose equivalent), total sugars (as sucrose equivalent), and minerals (iron and calcium) of control and best *Sel‐roti* were evaluated. The results of these evaluations are presented in Table 2.

**TABLE 2:** Nutritional comparison of control and 10% RSF incorporated *Sel‐roti*

Parameters	Control *Sel‐roti*	10% RSF incorpoated *Sel‐roti*
Moisture (%)	11.39 ± 0.25^b^	12.60 ± 0.32^a^
Crude fat (% DM)	27.31 ± 0.11^b^	28.75 ± 0.17^a^
Crude protein (% DM)	5.61 ± 0.08^b^	7.51 ± 0.11^a^
Total ash (% DM)	0.42 ± 0.03^b^	0.71 ± 0.04^a^
Crude fiber (% DM)	0.14 ± 0.01^b^	0.36 ± 0.04^a^
Carbohydrates (% DM)	66.52 ± 0.14^a^	62.66 ± 0.20^b^
Reducing sugar (% DM)	0.49 ± 0.02^a^	0.48 ± 0.02^a^
Total sugar (% DM)	16.46 ± 0.08^a^	16.35 ± 0.08^a^
Iron (mg/100g)	0.28 ± 0.02^b^	0.69 ± 0.03^a^
Calcium (mg/100g)	6.44 ± 0.12^b^	20.77 ± 0.17^a^
Energy value (Kcal/100g)	534.30 ± 0.67^b^	539.47 ± 1.13^a^

Note

Values are means of triplicates ± standard deviations. Values with the same superscript in a column are not significantly different (p > .05). (RSF: Roasted soy flour, DM = Dry matter).

#### 3.4.1 Moisture content

The moisture content of S*el‐roti* prepared by incorporation of 10% RSF was significantly higher (p < .05) than the control sample. This was possibly associated with high water adsorption characteristics of RSF as compared to rice flour (Agume et al., 2017; Chandra & Samsher, 2013). The moisture absorption and retention capacity of a product increase with the increase in its protein content (Jideani, 2011) and are affected by intrinsic factors of the product, which include size and shape of the protein, the hydrophilic‐hydrophobic balance of amino acids in the molecules, steric factors, and lipids and carbohydrates present in the flour (Acuña et al., 2012).

#### 3.4.2 Crude protein

The crude protein content of *Sel‐roti* prepared by incorporation of 10% RSF was significantly higher (p < .05) than the control sample. The high crude protein content of *Sel‐roti* prepared by incorporation of 10% RSF could be correlated to high protein content in soybean (42.23 ± 0.84) as compared to rice (7.18 ± 0.18) as shown in Table 1. A combination of lysine‐rich legumes and methionine‐rich cereal will yield a protein of high biological value (Balasubramanian et al., 2012). Roasting further enhances the digestibility of soybean protein (Agume et al., 2017). Therefore, the incorporation of roasted soy flour enhances the nutritional quality of *Sel‐roti* in terms of protein quality.

#### 3.4.3 Crude fat

The crude fat of *Sel‐roti* prepared by incorporation of 10% RSF was significantly higher (p < .05) than the control sample. The high crude fat content of *Sel‐roti* prepared by incorporation of 10% RSF could be partly correlated to the high‐fat content in soybean (16.45 ± 0.17) as compared to rice (1.46 ± 0.09) as shown in Table 1. However, the difference would be nominal at 10% RSF incorporation. Therefore, significantly higher (p < .05) crude fat content in the best sample was possibly associated with high oil adsorption characteristics of RSF (Agume et al., 2017; Chandra & Samsher, 2013; Twinomuhwezi et al., 2020) as compared to rice flour. The increase in the fat content of the product due to an increase in oil absorption of incorporated breadfruit flour on wheat flour was also reported in a fried doughnut (Oke et al., 2018).

#### 3.4.4 Total ash

The total ash content of *Sel‐roti* prepared by incorporation of 10% RSF was significantly higher (p < .05) than the control sample. The high total ash content of *Sel‐roti* prepared by incorporation of 10% RSF could be correlated to high total ash content in soybean (5.07 ± 0.06) as compared to rice (0.73 ± 0.04) as shown in Table 1. The increase in the ash content of product due to incorporated breadfruit flour on wheat flour was also reported fried doughnut (Oke et al., 2018).

#### 3.4.5 Crude fiber

The crude fiber content of *Sel‐roti* prepared by incorporation of 10% RSF was higher (p < .05) than the control sample. The high crude fiber content of *Sel‐roti* prepared by incorporation of 10% RSF could be correlated to the high crude fiber content in soybean (5.48 ± 0.08) as compared to rice (0.24 ± 0.03) as shown in Table 1. The increase in the fiber content of product due to incorporated breadfruit flour on wheat flour was also reported fried doughnut (Oke et al., 2018). Dietary fibers are attributed to the prevention of several diseases such as; cardiovascular diseases, diverticulosis, constipation, irritable colon, cancer, and diabetes (Coffin & Shaffer, 2006). Thus, the incorporation of soy flour in *Sel‐roti* could help minimize such cases.

#### 3.4.6 Carbohydrate, reducing sugar and total sugars

The carbohydrate content of the control sample was significantly higher (p < .05) than the *Sel‐roti* prepared by incorporation of 10% RSF. Carbohydrates content was calculated by the difference method and it was evident that high carbohydrates content in the control sample was due to its low content of other nutrients as shown in Table 1, namely crude fat, crude protein, total ash, and crude fiber. However, no statistical difference (p >.05) was observed between the samples in the contents of reducing sugars (as dextrose equivalent) and total sugars (as sucrose). This ensures comparable sweetness and the calorie from sugars between the samples.

#### 3.4.7 Minerals

The iron and calcium contents of samples were evaluated. The iron and calcium content of *Sel‐roti* prepared by incorporation of 10% RSF were significantly higher (p < .05) than the control sample. The higher contents in *Sel‐roti* prepared by incorporation of 10% RSF could be correlated to high contents of iron (7.36 ± 0.32) and calcium (221.78 ± 2.65) in soybean as compared to iron (0.45 ± 0.03) and calcium (11.07 ± 0.26) in rice as shown in Table 1. Enhancement of dietary calcium and iron has been recommended to women and children, the former has been attributed to skeletal health and the latter has been attributed to prevention of anemia (Lönnerdal, 2010). Thus, the incorporation of soy flour in *Sel‐roti* could also be helpful in the above aspects.

#### 3.4.8 Energy value

Energy value was calculated from the sum of the energy values for carbohydrates, protein, and fat by multiplying the contents of carbohydrates and proteins by 4 Kcal/g and the content of fat by 9 Kcal/g. The energy values of *Sel‐roti* prepared by the incorporation of 10% RSF were significantly higher (p < .05) than the control sample. The high energy content in best *Sel‐roti* was because of its high content, which has high energy density. Although a significant difference (p < .05) in energy value was observed between the samples, the numerical value difference was only 1%, which could be compensated by significantly enhanced (p < .05) sensory perceptions.

## 4 CONCLUSION

This study demonstrates that soy‐flour incorporation at levels up to 10% can enhance both the sensory and nutritional quality of *Sel‐roti*. The sensory perception scores of *Sel‐roti* significantly increased (p < .05) with an increase in levels of NSF up to 10% and significantly decreased at 15 and 20%. The organoleptic properties could be further enhanced if RSF or GSF could be used in place of NSF. The incorporation of 10% RSF resulted in the best sensory quality among the samples prepared in the study. The moisture, crude fat, crude protein, total ash, crude fiber, iron, calcium, and energy content of *Sel‐roti* could be significantly increased (p < .05) by the incorporation of 10% RSF. However, total carbohydrates was significantly reduced (p < .05) at 10% RSF incorporation. These findings may help to formulate a batter for the commercial production of *Sel‐roti* with enhanced sensory and nutritional properties. Further studies can be carried out on the storage stability of *Sel‐roti* to facilitate industrial application.

## CONFLICT OF INTEREST

No conflict of interest.

## AUTHOR CONTRIBUTION

**Bipana Thapa Magar:** Data curation (lead); Formal analysis (lead); Investigation (equal); Software (equal); Writing‐original draft (lead). **Surendra Bahadur Katawal:** Conceptualization (equal); Investigation (equal); Methodology (equal); Supervision (lead); Validation (equal). **Anuj Niroula:** Conceptualization (equal); Software (equal); Supervision (supporting); Validation (equal); Writing‐review & editing (lead).

## Supporting information

Table S1‐S2

## Data Availability

The data that support the findings of this study are available from the corresponding author upon reasonable request.
