# Supplementary material for: Enhancement of sensory and nutritional quality of Sel‐roti by the incorporation of soy flour
Source: Food Sci Nutr. 2021 Sep 16;9(11):6078–88. doi: 10.1002/fsn3.2550 (PMC8565246; doi:10.1002/fsn3.2550)
Supplement: Supplementary file 1 — Table S1‐S2 [file FSN3-9-6078-s001.docx]

**Supplementary table 1** Sensory perception scores of *Sel-roti* prepared by the incorporation of 0, 10 and 12.5% RSF

| **Parameters** | **Control (0% RSF)** | **10% RSF** | **12.5 % RSF** |
| --- | --- | --- | --- |
| Color and appearance | 6.3±0.47 ^c^ | 8.2±0.62 ^a^ | 7.6±0.50 ^b^ |
| Texture | 6.15±0.37 ^c^ | 8.05±0.51 ^a^ | 7.3±0.47 ^b^ |
| Flavor | 6.35±0.49 ^c^ | 8.1±0.55 ^a^ | 7.45±0.51 ^b^ |
| Taste | 6.3±0.47 ^c^ | 8.2±0.41 ^a^ | 7.45±0.51 ^b^ |
| Overall acceptance | 6.35±0.49 ^c^ | 8.15±0.37 ^a^ | 7.4±0.50 ^b^ |

Values are means of triplicates ± standard deviation. Values with the same superscript in a row are not significantly different (p > 0.05). (RSF: Roasted soy flour)

**Supplementary table 2** Physical properties of control (0% RSF) and 10% RSF incorporated *Sel-roti*

| **Parameters** | **0 % RSF** | **10% RSF** |
| --- | --- | --- |
| Weight per piece (g) | 39.32±0.87^a^ | 40.13±0.89^a^ |
| Ring diameter (cm) | 11.27±0.03^a^ | 11.24±0.03^a^ |
| Oil uptake (% DM) | 32.42±0.14^b^ | 33.87±0.23^a^ |
| Bulk density (kg/m^3^) | 386.5±2. 27^b^ | 408.61±2.33^a^ |

Values are means of triplicates ± standard deviation. Values with the same superscript in a column are not significantly different (p > 0.05). (RSF: Roasted soy flour, DM = Dry matter)
